# Dual activities of a silencing information regulator complex in yeast transcriptional regulation and DNA‐damage response

**DOI:** 10.1002/mlf2.12108

**Published:** 2024-05-15

**Authors:** Josephine Rybchuk, Wei Xiao

**Affiliations:** ^1^ Department of Biochemistry, Microbiology and Immunology University of Saskatchewan Saskatoon Saskatchewan Canada; ^2^ Toxicology Program University of Saskatchewan Saskatoon Saskatchewan Canada

**Keywords:** chromatin remodeling, DNA‐damage response, SIR complex, transcriptional silencing, yeast

## Abstract

The *Saccharomyces cerevisiae* silencing information regulator (SIR) complex contains up to four proteins, namely Sir1, Sir2, Sir3, and Sir4. While Sir2 encodes a NAD‐dependent histone deacetylase, other SIR proteins mainly function as structural and scaffold components through physical interaction with various proteins. The SIR complex displays different conformation and composition, including Sir2 homotrimer, Sir1‐4 heterotetramer, Sir2‐4 heterotrimer, and their derivatives, which recycle and relocate to different chromosomal regions. Major activities of the SIR complex are transcriptional silencing through chromosomal remodeling and modulation of DNA double‐strand‐break repair pathways. These activities allow the SIR complex to be involved in mating‐type maintenance and switching, telomere and subtelomere gene silencing, promotion of nonhomologous end joining, and inhibition of homologous recombination, as well as control of cell aging. This review explores the potential link between epigenetic regulation and DNA damage response conferred by the SIR complex under various conditions aiming at understanding its roles in balancing cell survival and genomic stability in response to internal and environmental stresses. As core activities of the SIR complex are highly conserved in eukaryotes from yeast to humans, knowledge obtained in the yeast may apply to mammalian Sirtuin homologs and related diseases.

## INTRODUCTION

*Saccharomyces cerevisiae* serves as a prime model organism in the study of eukaryotes and human health due to the highly conserved nature of genes and metabolic pathways. In *S. cerevisiae*, the silencing information regulator (SIR) complex transcriptionally represses bound loci through complex spreading and remodeling of the chromatin structure. The subcellular re‐localization and silencing activity of the SIR complex has implications in the regulation of metabolic gene expression, yeast mating‐type dictation, cell aging, and DNA damage response pathways. By understanding the multifunctional roles of the SIR complex as well as its individual proteins, one can better link critical pathways within cells and clarify eukaryotic aging or DNA‐damage response. Due to the similarity of yeast and human Sirtuin proteins, the findings in *S. cerevisiae* may ideally be extrapolated to higher‐level mammals, such as human health studies or aging theories. This literature review aims to investigate the functions of the budding yeast *S. cerevisiae* SIR proteins and their implications in various cellular pathways.

## THE SIR NETWORK

The *SIR* genes encode a silencing complex of SIR proteins, including Sir1, Sir2, Sir3, and Sir4. This complex was discovered in the late 1980s through mutations in previously known *STE8* and *STE9* genes, wherein further *sir* mutants complemented *sir1‐1* and *sir2‐1* but not *ste8* mutants, indicating the presence of a novel designated *SIR3* gene. Likewise, mutants of *sir* complemented *sir1‐1, sir2‐1*, and *sir3‐8*, but not *ste9*, indicating a novel *SIR4* gene^1^. Recruitment and binding of these proteins to DNA facilitates transcriptional silencing, wherein their localization is initiated via multiple signaling factors^2^. Transcriptional silencing occurs through protein–protein interactions between proteins at silent loci and SIR complex proteins. Upon recruitment and assembly of the silencing complex, deacetylation at histone tails occurs through Sir2 activity, allowing stabilization at bound loci and further spreading of the SIR complex, rendering bound regions inaccessible to replicative machinery^3^. Sir2 and higher eukaryotic Sirtuin proteins are deacetylases although some may remove malonyl or succinyl groups from target proteins^4^. There are seven mammalian Sirtuin proteins, where Sir2 most closely resembles mammalian SIRT1 with its histone deacetylase activity^4^, ^5^. Thus, data obtained on yeast Sir proteins ideally can be extrapolated to mammalian studies. Pathways in which the SIR complex is involved are well characterized in budding yeast, from their interactions in transcriptional silencing and chromatin remodeling, mating type dictation, to DNA damage response. The genes coding for NAD^+^‐dependent histone deacetylase Sirtuin proteins are essential for cellular regulation and survival as they are highly conserved from yeast to mammalian cells^6^.

Sir1 is regarded as a structural component in the SIR silencing complex binding to mating‐type loci. Sir1 was also shown to relocate to centromeres independently of Sir2, Sir3, or Sir4 through immunoprecipitation assays. In further chromosomal segregation assays, Sir1 was shown to play a vital role in chromosome stability through kinetochore interaction, where *sir1* null mutants had a staggering 21‐fold increase in chromosome loss, as measured through chromosomal missegregation rates and the demonstration that Sir1 interacts with centromere‐specific histone Cse4 and kinetochore protein Mcm19^7^. Beyond this recruitment to centromeres and promotion of heterochromatin formation, Sir1 is not shown to play a role in transcriptional silencing outside of the mating‐type loci complex formation and is not directly implicated in DNA damage response or aging^7^.

**Figure 1:**
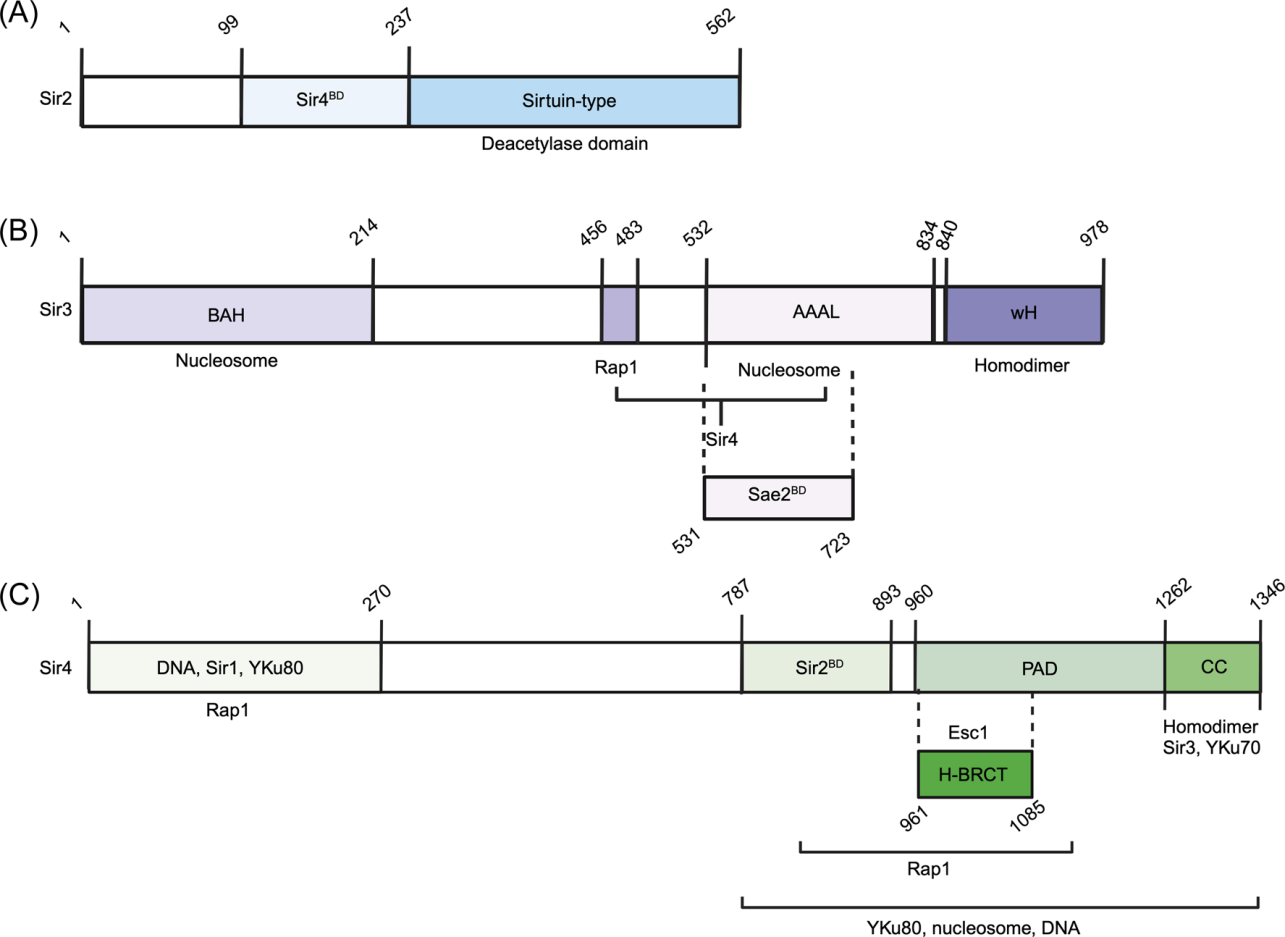
Domain structure of Sir proteins and their associated proteins. (A) The Sir2 structure with mapped Sir4 binding domain (Sir4^BD^, aa99–237) and a Sirtuin‐type deacetylase domain (aa237–562). (B) The Sir3 structure with a bromo‐adjacent homology (BAH) domain (aa1–214), a Rap1 binding domain (aa456–483), an ATPase‐like (AAAL)/nucleosome interacting domain (aa532–834), a winged‐helix (wH) domain (aa840–978) for Sir3 homodimerization, a Sae2 binding domain (aa531–723), and Sir4 interacting region (aa456–834). (C) The Sir4 structure. Its N‐terminal region (aa1–270) contains DNA‐binding, Sir1, and Yku80 interacting activities. A Sir2‐binding domain (Sir2^BD^) is mapped to aa787–893. It also contains a partitioning and anchoring domain (PAD) (aa960–1262) and a C‐terminal coiled‐coil (CC) domain (aa1262–1346) for Sir4 homodimerization. This C‐terminal CC domain is also required for physical interaction with Sir3 and Yku70. In addition, a noncanonical BRCA1 C‐terminal helix (H‐BRCT) functional unit is mapped to aa961–1085, which is also required for the Esc1 interaction. Rap1 interacting regions are mapped to two regions at aa1–270 and aa787–1262, and the C‐terminal aa787–1346 region is required for binding DNA and nucleosome, as well as Yku80. BRCA1, breast cancer susceptibility gene 1.

**Figure 2:**
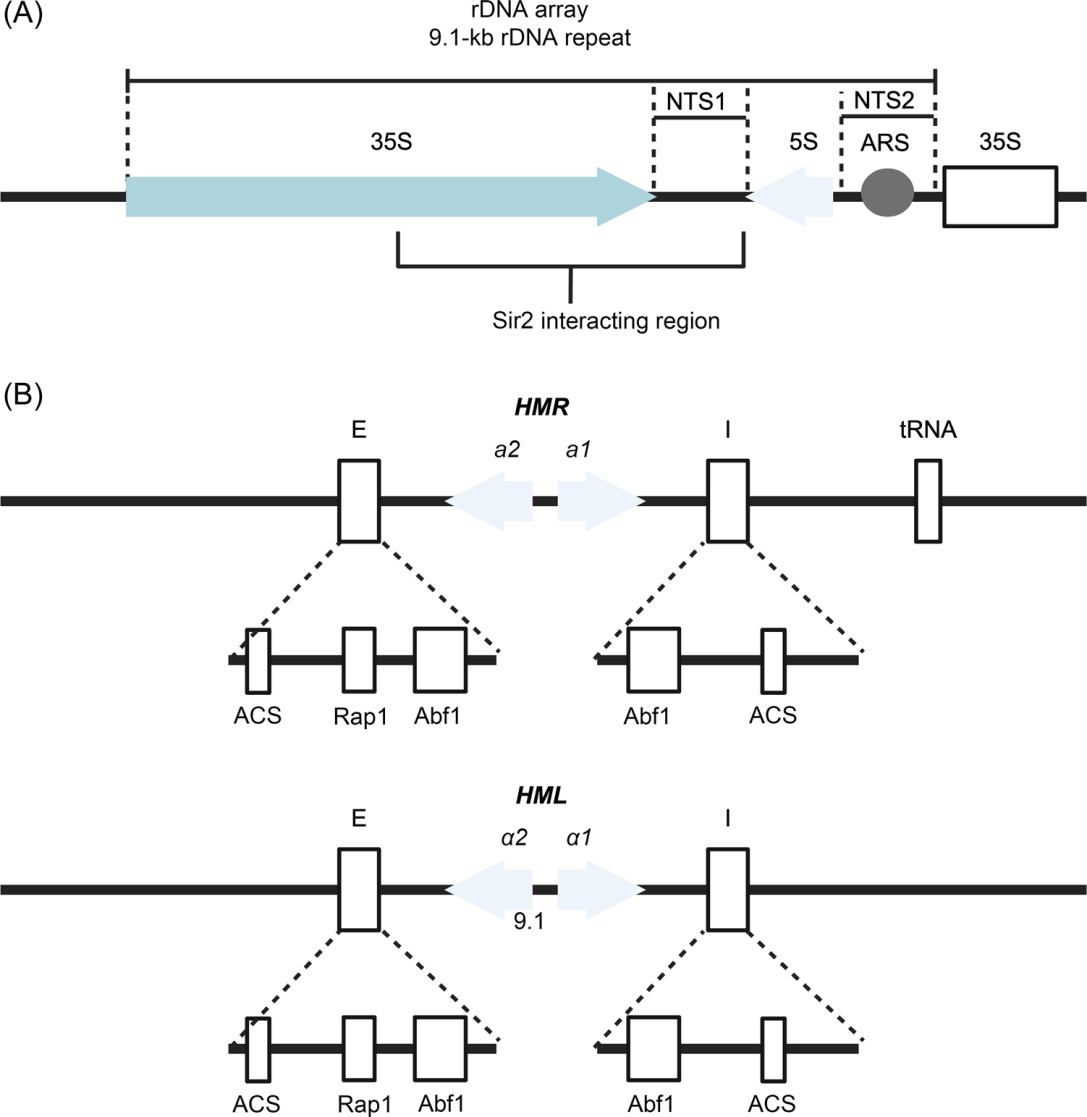
Chromosomal structures for a rDNA array and two mating‐type loci. (A) Chromosome XII contains approximately 150 copies of the rDNA repeats with one 9.1‐kb rDNA repeat consisting of 35S and 5S RNA coding genes, an autonomous replication sequence (ARS), and two nontranscribed spacer (NTS) regions 1 and 2. The mapped Sir2 interacting region is shown. (B) Each yeast *HMR* **a** and *HMLα* mating‐type locus contains two coding sequences, silencer regions E and I, two autonomically replicating consensus sites (ACSs), and Rap1 and Abf1 binding sites.

**Figure 3:**
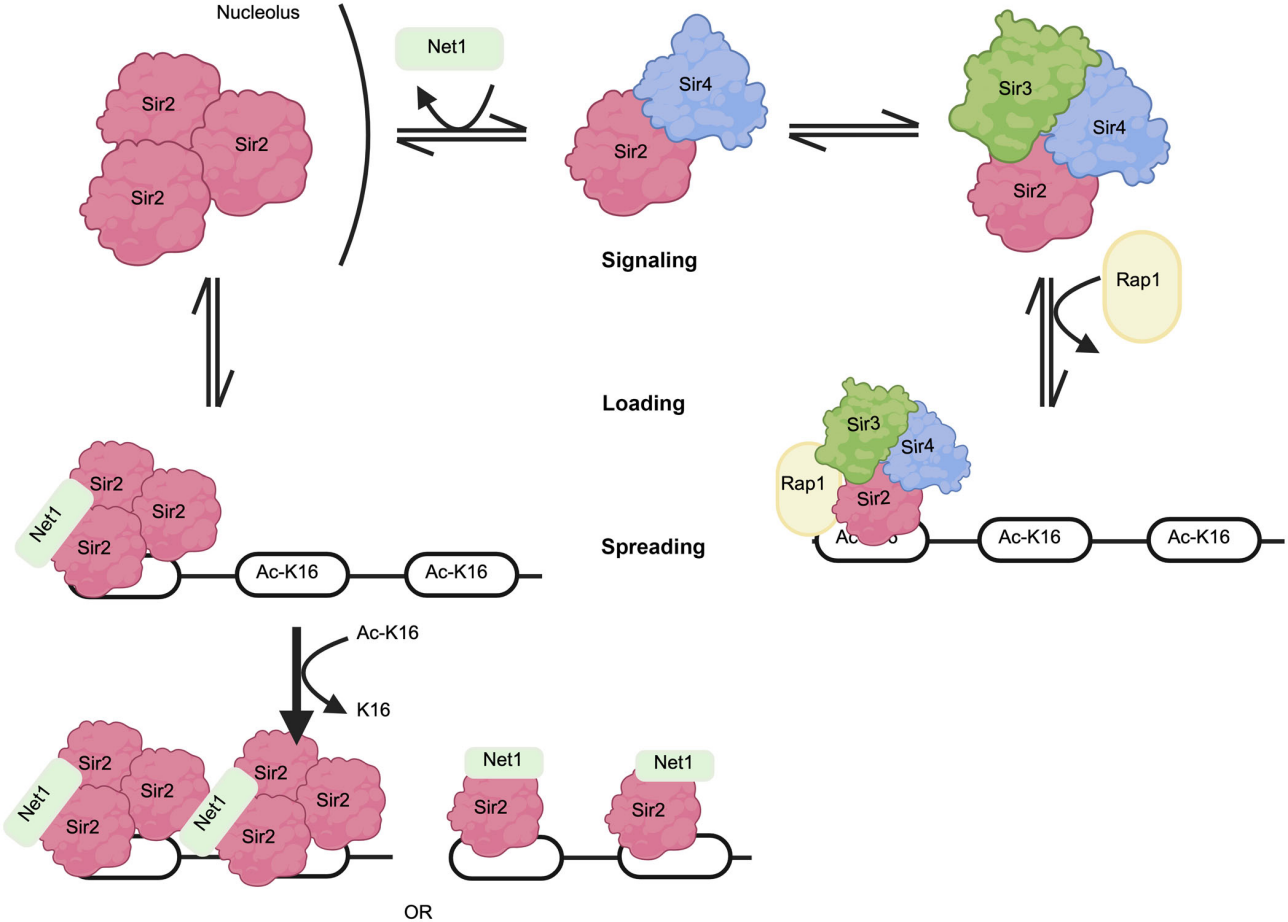
Diagram illustrating signaling events fostering the switch between Sir2 homotrimer formation and Sir2–Sir3–Sir4 heterotrimer formation between nucleolus and telomeres. Initially, Sir2 can dimerize with Sir4 and be further recruited to complex with Sir3. Upon trimeric complex formation, Rap1 signaling causes complex re‐localization to acetylated lysine residues on telomeric ends upon Rap1 interacting with Sir3. Alternately, Sir2 may dissociate from Sir4 and favor homotrimerization at nuclear‐acetylated lysine residues upon signaling from Net1. Upon homotrimerization or complex formation with Sir3 and Sir4, Sir2 deacetylates bound lysine and spreads along chromatin for transcriptional repression.

*SIR2* encodes a NAD^+^‐dependent deacetylase (Figure 1A) that targets lysine residues on proteins. The Sir2 deacetylase activity functions through a two‐step process: NAD cleavage to release nicotinamide, followed by substrate deacetylation^6^. Sir2 crystallography structure exploration revealed two independently folding domains consisting of an active C‐terminal catalytic domain with a NAD or ADP ribose binding site, and a helical N‐terminal domain. Key residue was identified for interdomain interaction leading to covalent binding of the C‐terminus and N‐terminus: the Arg235 residue that forms a salt bridge with Asp506, or two hydrogen‐bonds with Val490 and Glu504 carbonyl groups^8^. Sequence alignment of Sir2 proteins, such as Hst2, shows consistent conservation of the catalytic core domain for NAD binding. Substrates can bind Sir2 via both sides of the interdomain cleft, with a conserved protein tunnel within the cleft^9^. Using an *ADE2*‐*CAN1* dual selection and counter‐selection marker integrated into the rDNA array (Figure 2A), Sir2 was found to accumulate in rDNA regions, namely a major SIR‐responsive region in the nontranscribed spacer and a 35S rRNA coding region, to suppress transcription and reduce recombination via local structure remodeling and blocking access to further protein–protein interactions, independently of Sir1, Sir3, and Sir4^10^. This integration of the selectable marker *ADE2‐CAN1* into rDNA arrays allowed findings that *sir2* reduces expression of *ADE2* and *CAN1* in rDNA regions, where *sir1, sir3*, and *sir4* null mutants showed no difference in the expression of either *ADE2* or *CAN1* markers in rDNA^10^. Subsequent investigations indicated that Sir2 regulates recombination between different rDNA repeats instead of individual rRNA genes probably by forming special cohesin structures that inhibit unequal sister‐chromatid recombination^11^. It turns out that Sir2 contains a core deacetylation domain that facilitates the formation of a Sir2 homotrimer, which alone is sufficient for the transcriptional repression in rDNA. *sir2* null mutants were also shown to displace Mcm2–7 helicase from rDNA binding sites, shifting the localization of Mcm2–7 from a high nucleosome occupancy area to a low one, opposite of its traditional autonomous replication sequence (ARS)^12^. Mutations at conserved Sir2 residues in mice, yeast, or CobB (the *Escherichia coli* Sir2 homolog) strongly decreased Sir2 deacetylation activity on histones. Three classes of mutants were identified—one similar to wild‐type in vivo, two mutations that weakly reduced enzymatic activity for rDNA silencing, and the third class that entirely abolished rDNA silencing. Not only is deacetylase activity impacted, but experiments showed that particular mutations within this core Sir2 region also disrupted the localization of Sir2 homocomplexes and Sir2–Sir4 complex formation^13^. On the other hand, preferential binding of Sir4 to Sir2's N‐terminus (Figure 1A) is thought to inhibit the formation of the Sir2 homotrimer and instead favors the formation of the heterotrimeric Sir2–Sir3–Sir4 in chromatin silencing at homothallic silent loci in yeast^14^ (Figure 3). *SIR2* is also essential for moderating recombination in yeast cells where *sir2* null mutants showed a notable uptick in recombination efficiency^15^. This is thought to be a result of Sir2 localizing at double‐strand breaks (DSBs) or rDNA to prevent homologous recombination (HR) by binding free DNA ends^16^. Lastly, Sir2 may play a role in cytoplasmic pH regulation for *S. cerevisiae* via Pma1, a P‐type H^+^‐ATPase, upon signaling from environmental factors such as nutrition or growth signals. Sir2 phosphorylation was shown to influence *PMA1* expression via the TORC1 signaling pathway, associating it with cell growth and yeast replicative lifespan (RLS)^17^.

Sir3 (Figure 1B) is a key complex structural component recruited upon initial binding of Rap1 and Sir2, where it functions with Sir4 to allow the spreading of the silencing complex along chromatin (Figure 3). Through analysis of the Sir3 crystal structure, three key regions were identified: an N‐terminal arm structure conducive to oligomerization, a canonical AAAL domain lacking nucleotide binding capability, and a four‐helix bundle within a Sir3 subdomain^18^. Although Sir3 binding occurs primarily in its C‐terminus, a critical bromo‐adjacent homology (BAH) domain in its N‐terminus is required for silencing. The Sir3 BAH domain is shown to bind the H3 nucleolar exposed region of H3K79 and the N‐terminal region of H4K16, as well as further interact with the remaining histones comprising the four core histones due to the negative charge of the binding pocket^19^, ^20^. Sir3 also contains an AAAL domain that interacts with nucleosomes and the Sir4 coiled‐coil (CC) domain in a methylation‐sensitive manner at H3K79, where mutations within this AAAL domain nullify silencing. A key loop within the AAAL domain is required for Sir4 interaction^18^, ^20^, ^21^. Lastly, Sir3 contains a winged‐helix (wH) domain at its C‐terminus, contributing to nucleosome binding as well as Sir3 dimerization for silencing^2^. Beyond being a component of chromatin silencing, Sir3 is also implicated in DNA damage response by binding next to DSBs upon signaling from checkpoint proteins Mec1 and Rad9^22^, and through physical interaction with Sae2 and inhibition of HR^23^.

Sir4 is thought to serve as a scaffold protein in the SIR complex formation and functions via a Sir2‐interacting domain and Sir3‐interacting domain (Figure 1C). Sir4 localization requires its RKR residues within its breast cancer susceptibility gene 1 (BRCA1) C‐terminal helix (H‐BRCT) domain, allowing for subtelomeric silencing at phosphorylated residues. Mutations at the RKR site disrupt traditional phospho‐peptide interactions and reduce the formation of Sir4 foci within the nucleus and at telomeres. This H‐BRCT domain functions as an active unit in the Sir4 partitioning‐and‐anchoring (PAD) domain^24^ (Figure 1C). The Sir4 CC domain at the C‐terminus enables both Sir3 and yKu70 interactions, as well as Sir3 dimerization, where the CC domain is sufficient for homothallic mating‐type (*HM* locus silencing but not necessarily telomeric silencing^24^. This silencing occurs through the Sir4–Esc1 interaction at phosphorylated motifs via Sir4's C‐terminal PAD domain, or preferential binding to Sir2 during heterocomplex formation (Figure 1C). Esc1, upon binding Sir4, serves as a localization factor through telomeric partitioning of Sir proteins^24^, ^25^, ^26^. Sir4 facilitates telomeric silencing by interacting with Dot4 and Ubp3 ubiquitin hydrolases^27^. Sir4 interaction with Sir2 enhances Sir2's catalytic activity, particularly with respect to mitotic progression^28^. More recently, Sir4 is shown to repress subtelomeric recombination through inhibition of subtelomeric Y' element activity upon complexing with Sir3 and re‐localization to subtelomeric elements^29^. Nucleoporin Nup170 is traditionally shown to cluster at subtelomeric sites for telomeric silencing and tethering to the nuclear envelope, where Sir4 is shown to interact with and mediate Nup170 binding to subtelomeric chromatin. The Sir4–Nup170 complex in association with Esc1 is required for loading to subtelomeric chromatin and preservation of chromatin integrity, where loss of one protein results in disruption of loading and silencing for the complex^30^. Interestingly, the Sir4 C‐terminal structure mimics the human nuclear Lamin A and C central rods, suggesting a relation between Sir4 and conserved nuclear lamina structure, making it possible that Sir4 mediated silencing at silent mating type loci is due to their association with yeast nuclear lamina^31^.

Altogether, these proteins are essential for silencing yeast chromatin due to their chromatin remodeling activity. As a complex, Sir2–4 works to remodel chromatin into a transcriptionally silent heterochromatic state^32^. They are implicated in multiple pathways, from mating‐type gene expression to DNA damage response, and are believed to be indelible to cell survival. They serve as a key model for better understanding such vital processes in cells, as they are conserved with characterized mammalian homologs of the Sirtuin proteins.

## SIR IN MATING‐TYPE GENE REGULATION

Budding yeast *S. cerevisiae* may alternate between haploid mating types *MAT* **a** and *MATα* during its life cycle^33^. Although typically, the switch between mating types is done through cellular fusion to cells of the opposite mating type, haploid cells may carry out the switch individually^27^. The evolutionary significance of mating‐type switching is clear, as many yeast species besides *S. cerevisiae* also demonstrate the alternation, where switching can be inferred to confer an advantage for host survival^34^. The expression of either mating type is controlled by repression at the *HM* locus containing the mating‐type information sequences, *HMLα* and *HMR* **a** (Figure 2B). The presence or absence of a silencing complex at *HM* loci dictates which mating‐type cassette information is expressed at the *MAT* locus. In *MAT* **a** cells, ***a**1* and ***a**2* genes are expressed, while in *MATα* cells, *α1* and *α2* are expressed^35^.

Interconversion between mating types in homothallic yeast is initiated by an *HO* site‐specific endonuclease activity. Binding sites at either side of silencing loci for Rap1, Abf1, and origin recognition complex (ORC) are necessary for the SIR complex recruitment. Upon binding, the SIR complex deacetylates various lysine residues at histones H3 and H4, wherein the deacetylation of Lys16 (K16) in H4 is crucial for silencing at mating‐type loci^36^. Upon SIR complex spreading, *MAT* **a** seeks *HMLα*, or *MATα* seeks *HMR* **a** to switch mating types via an HR‐mimicking reaction. This mating‐type switch may serve as an evolutionary advantage by improving cell survival^37^. As cells age, accumulated mutations in *SIR4* have been linked to shortened lifespan and a new mating‐type phenotype. Upon loss‐of‐function *SIR4* mutations, there is de‐repression at the *HM* loci and expression of an **a**/*α* phenotype despite the cell's haploid state. Although the de‐repression at mating‐type loci does not outright cause cell mortality, these cells display sterility^38^, which prevents them from entering the sexual cell cycle.

The tight regulation of mating type expression in yeast by the SIR silencing complex has implications beyond **a** */α* characteristics and advantageous reproduction. In fact, homozygous diploids show rescue of cell survival in the presence of DNA‐damaging agents in comparison to haploid cells. Thus, the signaling behind SIR complex recruitment and silencing may be related to the suppression of DNA repair in aging cells or some other moderation of pathway signaling upon lesion formation^39^.

Mating type is shown to impact repair pathways in yeast, where haploid cells favor nonhomologous end joining (NHEJ) while diploid cells use existing homologous sequences to perform HR. In haploid cells containing *sir* mutations, cells behaved as diploid despite the absence of mating and preferentially induced HR^40^.

## SIR IN SUBTELOMERIC GENE SILENCING

Beyond binding to *HMR* or *HML* silencing loci, Sir proteins may be recruited to subtelomeric regions upon Rap1 binding. Rap1 binds Sir3 and Sir4 directly (Figure 1B,C); however, the initial binding event is between Rap1 and Sir3 at telomeric DNA^41^. The binding and formation of the SIR silencing complex blocks binding sites for transcription factors at silencing enhancers, leading to transcriptional repression. This clustering of Sir proteins and transcriptional repression is called the telomere position effect (TPE) and helps promote cell stability^2^. Generally, TPE absolutely requires Sir2, Sir3, Sir4, Rap1 C‐terminus, or an NHEJ protein complex Yku70/80 for silencing^42^, ^43^, ^44^, whereas Sir1 is dispensable^45^. The extent of the TPE can be dictated by histone acetylation levels resulting from the Sir2 deacetylase activity, affording protection of telomeric ends from degradation^46^. Alternately, the Sir4 N‐terminal domain participates in the TPE upon binding yeast Ku proteins Yku70 and Yku80, thus recruiting other Sir proteins^47^.

Together, the yeast SIR complex functions in silencing at *HM* loci and subtelomeric regions through crosslinking and compacting chromatin fibers along nucleosomes, beginning at H3 and H4 lysine residues^48^. This transition from transcriptionally active euchromatin to transcriptionally repressed heterochromatin is in part fostered by the SIR complex interaction with nuclear envelope‐bound anchoring proteins during silencing. When the nuclear envelope structure is distorted, Sir4 interaction with telomeric regions and subsequent hyperclustering is disrupted, resulting in loss of transcriptional silencing. A recent study investigated the impact of Sir4/telomere interaction disruption and found that ribosomal protein gene expression was notably downregulated due to accumulative rDNA compaction^49^. The telomeric silencing by Sir proteins is shown to be dependent on nuclear envelope lipid content, where links between chromatin remodeling and lipid metabolism have been previously established. Modification of the nuclear envelope altered telomeric SIR clustering and resulted in a partial loss of complex silencing at subtelomeric genes^49^.

Sir2, shown to bind multiple yeast silent loci, also plays a role in subtelomeric silencing. Some telomeric genes are shown to have Sir2 binding domains where Sir2‐dependent silencing occurs in a distinguishable manner to alternative Sir2‐independent silencing^50^. *SIR2* binding represses mitotic recombination and transcription of a marker gene at rDNA arrays, where Sir3 and Sir4 proteins also relocate from telomeres to the nucleolus via *UTH4* and *YGL023* in an age‐dependent manner, suggesting that rDNA may serve as an *AGE* locus^51^.

## SIR AND DSB REPAIR

Aside from silencing at *HM* loci and subtelomeric regions, studies have shown that Sir proteins participate in signaling upon DNA damage by directing between HR and NHEJ pathways. In budding yeast, HR is coordinated by the *RAD52* epistasis group genes^52^, ^53^, while NHEJ requires a Ku heterodimer consisting of Yku70 and Yku80^54^, yet the link between the two seems mediated by the SIR complex^40^. In fact, *sir2, sir3*, and *sir4* mutations—but not *sir1*—were shown to reduce NHEJ almost to the same extent as *yku* mutations^55^. Thus, Sir protein binding and complex activity must play a crucial role in fostering the end‐joining of DSBs, preferentially inducing NHEJ over HR.

In budding yeast, NHEJ is initiated when Yku70/80 and Mre11/Rad50/Xrs2 (MRX) bind the DSB ends to repair them through endo‐ and exo‐nuclease activities^56^. It is Yku activity that associates more closely with the SIR complex, where cells deficient in Yku had increased DNA damage sensitivity related to NHEJ. In fact, Yku80 binding to internal chromosomal sequences requires the SIR complex, although its telomeric binding is SIR independent^57^. NHEJ reduction is observed in *sir*Δ mutants, where it is thought to be due to the de‐repression of *HM* loci since haploid cells retained NHEJ, whereas diploid cells preferentially underwent HR. Nonetheless, Sir proteins play a more minor role than Yku proteins in NHEJ, where the SIR complex simply organizes DNA ends at DSBs through heterochromatic structure formation upon Sir4 interaction with Yku70 or Yku80^40^. The connection to mating type can be further clarified where a gene required for NHEJ is regulated by the **a**1/*α*2 repressor. NHEJ efficiency or repression could be controlled by the presence of haploid or diploid mating‐type traits. With diploid yeast containing **a**/*α*, the homology‐directed repair is preferred as an adjacent undamaged template may be used, while haploid strains may favor NHEJ over HR. Thus, NHEJ preference is signaled by mating‐type heterozygosity^58^.

This redirection can be further clarified by studying Sir protein functions individually in DNA damage response. Upon DNA damage signaling by Mec1 and Rad9, Sir3 dissociates from telomeres to relocate to DSB sites during S‐phase^22^. Sir3 is responsible for diverting HR upon propagating cis repression at DSBs and trans repression by binding Sae2^23^. It is this binding of Sae2 by Sir3 that prevents HR, as HR is initiated by Sae2/MRX binding to and processing DSB ends while preventing binding of other factors for NHEJ^59^, ^60^. Sir4 is also necessary to promote NHEJ by binding telomeric ends, inhibiting degradation, and recruiting telomerase through Rap1 interaction independently of Sir2 and Sir3 binding^61^. Together, Sir3 and Sir4 foster Yku complex binding through competitive inhibition of Sae2‐MRX activity and promotion of DNA integrity until NHEJ is initiated. Sir4 recruitment to telomeres is mediated by sumoylation via a SUMO E3 ligase Siz2, which also sumoylates Yku70/80. This Siz2 activity not only recruits essential proteins for NHEJ but helps to localize proteins to telomeric ends and DSBs^62^. Upon Sir4 movement, Sir4 interaction with Esc1 is believed to anchor the chromatin while a Sir4^PAD^ domain directly interacts with Yku80^63^. Thus, upon Sir3 competitive inhibition of Sae2‐MRX, Sir4 binding of Esc1 and Yku80 is thought to further the repressive effect on chromatin and promote NHEJ.

## SIR AND DNA POSTREPLICATION REPAIR (PRR)

Diploid cells heterozygous for mating type (**a**/α) are more resistant to ultraviolet (UV) irradiation and display higher‐level recombination than haploid cells or diploids homozygous for mating type genes^33^, ^64^. These differential levels of DNA‐damage sensitivity between haploids and heterozygous diploids have been shown in cells defective in *rev3* ^65^ or *rad18* ^66^, which were abolished when combined with mutations involved in recombination repair^67^, suggesting that UV‐induced lesions in these mutants are channelled to HR repair.

The mating‐type heterozygosity or pseudodiploid can be created by transforming haploid cells with a YCp plasmid carrying opposite mating‐type genes, while the same cells carrying the same mating‐type genes serve as a control. Indeed, it was found that mating‐type heterozygosity rescued *RAD6* DNA PRR pathway mutants but not other DNA repair pathway mutants from killed by a variety of DNA‐damaging agents and that this rescue effect was dependent on functional HR but independent of NHEJ^39^. PRR was first defined in budding yeast as a cellular activity to fill in UV‐induced single‐strand DNA (ssDNA) gaps^68^. Since this activity does not remove DNA adducts but rather promotes survival in the presence of DNA damage, it is renamed as a DNA‐damage tolerance (DDT) mechanism^69^. When encountering a replication‐blocking lesion, budding yeast replicative DNA polymerase δ (Polδ) is stalled, leading to proliferating cell nuclear antigen (PCNA) monoubiquitination by an E2–E3 complex Rad6‐Rad18 at its K164 residue. Monoubiquitinated PCNA recruits translesion DNA synthesis (TLS) polymerases Rev1, Polη, and Polζ to replace Polδ and bypass the lesion with (Rev1 and Polζ) or without (Polη) increased mutations. The monoubiquitinated PCNA can be further polyubiquitinated by another E2–E3 complex Mms2/Ubc13‐Rad5 through a noncanonical K63‐linked Ub chain to promote error‐free lesion bypass by using the newly synthesized sister chromatid as a template^70^, ^71^. Furthermore, in the absence of DNA damage, PCNA‐K164 can be modified by a small Ub‐like modifier (SUMO), which is mediated by a SUMO E2–E3 complex Ubc9‐Siz1^72^, ^73^ and leads to the recruitment of a DNA helicase Srs2 to prevent salvage HR^74^, ^75^. Srs2 is a 3′–5′ DNA helicase that inhibits the Rad51‐ssDNA filament formation^76^, ^77^, and rescue of *rad6* and *rad18* DNA damage sensitivity by inactivation of *SRS2* relies on functional HR^78^.

Silencing at the mating type loci is determined by cis‐acting regulatory silencer sequences: *HML‐E*, *HML‐I*, *HMR‐E*, and *HMR‐I* ^79^. Such elements can interact with which trans‐acting factors including Sir proteins, Rap1, histones H3 and H4. These direct or indirect interactions establish or maintain silencing at mating‐type loci^45^, ^80^, ^81^. Hence, the budding yeast pseudodiploid state could also be created by inactivating the *SIR* genes since the *SIR* gene mutation allows simultaneous expression of both mating‐type loci. Indeed, a genetic screen utilizing a PRR pathway mutants identified a mutation in *SIR3* ^39^, although how this mutation affects mating‐type heterozygosity and DNA‐damage response remains unclear.

## SIR AND AGING

*S. cerevisiae* historically has served as a model for aging studies, with two methods of observable age progression. One is RLS, where a single mother cell is studied through its budding generations. After around 23 generations, the mother cell becomes much larger than the starting size before ultimate cell granulation or lysis. Mother cells have longer generation time, than their produced daughter cells. This is probably due to cell senescence‐related factors expressed in the cytoplasm by mother cells that may be transmitted to daughter cells but are diluted through generations. Mother cells appear to continuously generate this cytoplasmic factor, while daughter cells merely respond to it. Thus, mother cells have longer generation time during cell senescence^38^, ^51^, ^82^.

Another method is chronological lifespan (CLS), or studies of the whole yeast population, which can be observed via chitin/bud scarring accumulation. This observable buildup is due to daughter cell separation and cell wall repair in the mother cell^83^, ^84^, ^85^, thus providing a way to visualize yeast aging. Rather than studying cell viability during and out of reproduction, replicative aging studies the length of time a cell can maintain division based on replicative fitness. Over time, mature mother cells become sterile due to a variety of signaling factors, such as cell damage, thus losing the initiation of meiosis or mitosis^86^, ^87^. Life‐extending mutations are shown to play a role in RLS extension in yeast, thus highlighting the difference between chronological and replicative aging aspects of yeast^86^. In investigating yeast aging, *SIR* genes have been shown to form a part of the epigenetic mechanism for aging. The link between the SIR complex, yeast sterility, and aging has been characterized as early as the late 1990s^38^, where a loss of silencing at *HM* loci resulted in sterile **a**/*α* cells and cell mortality. This loss of silencing complex activity was further elucidated by studying *sir* null mutants, wherein some *sir* null mutants had an extended lifespan and lacked sterility. This novel age‐related phenotype could be visualized through examination of telomere composition in sterile, aged yeast cells. When studying telomere length based on analysis of bud scars, *sir3* and *sir4* null mutants showed a shortening of telomeres compared to wild‐type cells, thus a decrease in RLS. This study only further implicates the SIR complex in yeast aging processes, particularly Sir3 and Sir4 in this case. The gain of sterility is thought to be due to the re‐localization of the SIR complex to unstable rDNA, as part of aging linked to rDNA accumulation^51^, ^88^. This characterization is key, as the SIR complex alters histone acetylation, and histone overexpression or total histone levels versus tight regulation of histone acetylation levels has been linked to changes in lifespan^89^, ^90^. Nucleosome structure dictates transcription levels, and acetylation of histone tails at lysine residues controls the accessibility of chromatin, wherein alteration of acetylation states through deacetylation and spreading of a silencing complex along the chromatin represses transcription^2^.

Acetylation of histones and their association with aging are thought to be in part modulated by Sir2. Histone acetylation is critical as null mutants involved in histone expression, such as *asf1* or *sir2*, have dramatically shortened lifespan compared to wild‐type cells, while overexpression of histone‐associated proteins increases lifespan^89^. In fact, Sir2 blocks CLS extension where *sir2*Δ mutants in combination with a serine/threonine protein kinase gene mutation *sch9*Δ extended lifespan. Alternatively, yeast *SIR2* overexpression resulted in an extension of yeast RLS^91^. In comparison, the antisilencing gene *ASF1*, also required for histone H3 and H4 acetylation, integrity, and gene expression regulation, has a shorter lifespan when it is deleted compared to *sir2*Δ mutants^89^. Since the *asf1 sir2* double mutant is more sensitive than their respective single mutants, *ASF1* and *SIR2* may function in separate pathways in aging processes.

These findings in yeast hold the key to understanding higher eukaryotic systems. In mammalian cells, Sir2 homologs SIRT1, SIRT2, SIRT3, and SIRT6 similarly deacetylate histones. As Sirtuins are conserved through all eukaryotes, yeast Sirtuins may provide keen insight into aging by studying Sir protein levels during cell cycles. Sirtuins are essential for many physiological processes and have been linked to carcinogenesis, apoptosis, cell survival, pathophysiologies such as kidney disease, DNA repair, viral replication, and cell senescence^92^. Yeast Sirtuins, particularly Sir2, have been shown to regulate metabolic processes such as glycolysis and further metabolic intermediary protein modification^93^. Hence, studying orthologous yeast Sirtuin pathways can reveal key insights into mammalian disease processes and cellular aging. Yeast DNA repair pathways may even be interwoven with cellular aging due to their alteration of the epigenetic landscape and an increase in genomic instability due to genetic upregulation and loss of heterozygosity^94^, ^95^.

A previous study of the role of Sir2 in aging linked its activity at subtelomeric regions where H4K16 acetylation decreased, correlating with a decrease in lifespan. Overexpression of *SIR2* rescued cell lifespan beyond wild‐type length^96^. In fact, Sir2 alone is shown to have two separate functions with telomere length regulation and senescence of yeast mother cells. While a decrease in H4K16 acetylation is associated with senescence progression, Sir2 telomeric localization is linked to RLS through reduction of rDNA recombination and formation of extra‐chromosomal rDNA circles (ERCs)^51^, ^93^. Sir3 and Sir4 may also prevent aging by re‐localizing to the nucleolus to either prevent DNA damage accumulations or promote DNA repair. *SIR3* overexpression causes telomeric hyperclusters, counteracting telomeric anchoring via a Sir4 interaction with the Sir3 C‐terminus. This clustering localizes silencing at the telomeric ends in a manner independently of *HM* silencing. In aging, the recruitment of Sir3 to bridge subtelomeric and rDNA structures through array formation can be seen^97^. The Sir3‐associated telomeric hyperclustering is induced upon binding Esc1, which localizes hyperclusters to the nuclear membrane in a Yku‐independent manner. This hyperclustering is more associated with cellular quiescence but is not required for cell survival, thus casting some confusion on the role of Sir3 in aging^98^. Studies suggest that this Sir3‐directed localization of hyperclusters fosters long‐term cell survival during quiescence, although Sir3 activity is not required for establishing quiescence specifically^99^.

The role of Sir4 in aging is even less understood; however, a *sir4‐42* mutant had a dramatically increased lifespan. The gain‐of‐function mutation requires functional Sir2 and Sir3, while it causes yeast sterility as well as a loss of *HM* locus and telomeric silencing^100^. This *sir4‐42* mutation removes 121 amino acid residues from the C‐terminus and was found to re‐localize itself and Sir3 from *HM* loci and telomers to a proposed *AGE* locus. Indeed, the *sir4‐42* mutant phenotype could be recapitulated by expressing the C‐terminal coding region of *SIR4* ^100^. Subsequent studies identified the *AGE* locus to be rDNA within the nucleolus^51^. Although the mechanism of this pathway remains elusive, Sir3 and Sir4 activities are directed by *UTH4/MPT5*, a gene also associated with yeast aging^51^. Additionally, Sir4 has been shown to bind Sir3 and inhibit subtelomeric recombination during cell senescence; disruption of *SIR4* allowed for Y′ element telomere elongation and rescued cell senescence^51^.

Despite Sir3 and Sir4 correlations with aging and *HM* locus de‐repression, it is undoubtedly that Sir2 plays the more crucial role in lifespan moderation, particularly during times of caloric deprivation or environmental stress^16^, ^93^. Recent findings suggest that beyond maintenance of chromosomal integrity and telomeric protection, Sir2, through interaction with either the TORC1 and/or the cAMP/PKA pathways, can moderate cell growth depending on glucose availability^17^.

Although more research is necessary to fully clarify the role of *SIR* genes in aging, many preliminary links and functions have been established thus far, highlighting their important roles in the process.

## YEAST SIR AND HUMAN SIRTUINS

Yeast is an excellent model for studying evolutionarily conserved eukaryotic genes, as evidenced above, and findings in yeast Sir proteins have been translated over to their human orthologues, including SIRT1, SIRT2, and SIRT3. SIRT1 is a TATA box deacetylase, promoting transcriptional repression, as well as showing multiple other transcription or protein modification activities such as p53 deacetylation, which has links to preventing tumorigenesis and maintenance of normal cell metabolism^101^. Beyond moderation of key metabolic genes, the SIRT1 activity is implicated in other pathological and physiological conditions such as diabetes, obesity, Alzheimer's, embryogenesis, and lifespan^102^, ^103^, ^104^, ^105^. Understanding SIRT1's direct orthologue, Sir2, may provide key insights into the aforementioned pathways and lend us a better understanding of the development and treatment of various human pathophysiology.

SIRT2, another Sir2 orthologue that more closely mimics yeast Hst2, is responsible for microtubule remodeling for transcriptional repression. Tubulin remodeling by SIRT2 and HDAC6 plays a role in cell shape, intracellular signaling, cell motility, and traditional cell division, leading to potential health implications if tubulin acetylation/de‐acetylation is not tightly regulated^106^. Using *S. cerevisiae* Sir2 and Hst2 as models, we can better understand the binding and deacetylase activity of SIRT2 in normal cell processes.

Lastly, SIRT3, the yeast Sir2 homolog, is implicated in human metabolic pathways upon caloric restriction, leading to upregulation of adipocyte metabolism. Beyond metabolic functions, human SIRT3 is involved in moderating nuclear membrane potential and protection against the formation of toxic reactive oxygen species (ROS) through increased oxygen consumption^107^. SIRT3 interacts with FOXO3a and contributes to genetic longevity via induction of FOXO3a‐dependent gene expression and reduction of ROS production^108^. Additionally, during glucose deprivation, induced SIRT3 in mitochondria plays a critical role in sustaining neurotransmission plasticity^109^. Hence, understanding Sir2 can further tie together the linkage of SIRT1 and SIRT3 to obesity or other metabolic‐related disorders by studying both expression and activity levels of the related proteins as compared to yeast Sir2.

In summary, the conservation of yeast Sir2 NAD^+^‐dependent deacetylase activity in its human counterparts SIRT1, SIRT2, and SIRT3 indicates the importance of Sirtuin proteins in maintaining essential cell processes. During studies of human pathophysiology by understanding its causes and impacts on health and the development of treatment methodologies, one can look back to the yeast Sir2 model in improving our understanding of human Sirtuin contributions to our health.

## CONCLUDING REMARKS

**Figure 4:**
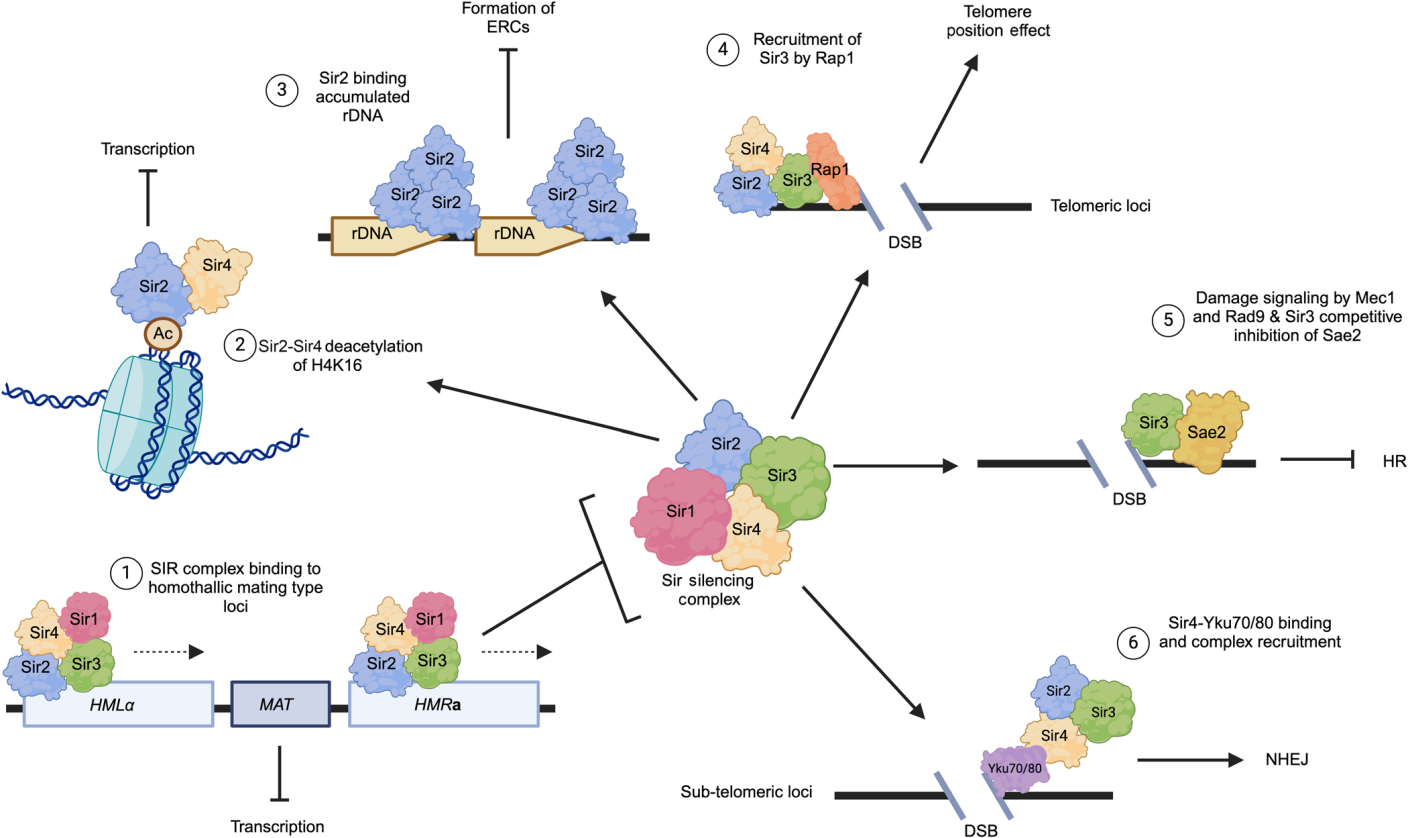
Dual activities of the silencing information regulator (SIR) complex and the resultant functions. (1) The SIR complex is recruited to mating‐type loci at consensus sequences within silencers, where Sir2 deacetylates bound lysine residues and the complex spreads along the chromatin at those loci to repress transcription, allowing expression of the opposite mating type at the *MAT* locus. (2) Sir2–Sir4 are recruited to acetylated histone tails to deacetylate H4K16 and repress transcription. (3) Sir2 binds to Sir2 responsive regions at rDNA arrays at telomeric or non‐telomeric ends, preventing the formation of extra‐ribosomal circles (ERCs) and chromosomal degradation. (4) Recruitment of Sir3 by Rap1 to double‐strand break (DSB) regions and subsequent complex formation with Sir2 and Sir4. Complex formation leads to the reorganization of chromatin and the telomere position effect, promoting DSB resection and repair. (5) The recruitment of Sir3 to DSB ends upon signaling from Mec1 and/or Rad9 and the competitive inhibition of Sae2, blocking homologous recombination (HR) at free DSB ends. (6) Initial Sir4 binding to Yku70/80 around free DSB ends before recruitment of Sir2 and Sir3 for the SIR complex formation, which is followed by Yku70/80‐mediated nonhomologous end joining (NHEJ) at the damaged region.

Sir proteins appear to possess two major activities, namely transcriptional silencing at multiple chromosomal locations through histone acetylation and chromosomal remodeling and modulation of DSB repair pathways through physical interaction with proteins involved in HR and NHEJ. These activities either act alone or in combination to influence multiple cellular pathways involved in epigenetic regulation and DNA damage response, leading to altered cell viability and death, as summarized in Figure 4. Sir proteins may hold the key to understanding vital physiological processes in cells, from genomic stability to epigenetic aging. Ongoing research continues to further the association of *SIR* genes and their role in ensuring cell survival through these pathways, as a number of key questions outlined in this review, have not been fully addressed. Due to their highly conserved nature, observations made in yeast Sirtuin homologs may be extrapolated to higher eukaryotes, allowing us to guide similar research in mammalian cellular processes. Only time and further work will better define those roles, which hold major implications for our understanding of vital human cellular mechanisms for survival and programmed aging.
